# Techno-economic analysis of the deacetylation and disk refining process: characterizing the effect of refining energy and enzyme usage on minimum sugar selling price and minimum ethanol selling price

**DOI:** 10.1186/s13068-015-0358-0

**Published:** 2015-10-29

**Authors:** Xiaowen Chen, Joseph Shekiro, Thomas Pschorn, Marc Sabourin, Melvin P. Tucker, Ling Tao

**Affiliations:** National Bioenergy Center, National Renewable Energy Lab, 1617 Cole Blvd, Golden, CO 80127 USA; Andritz Inc., 3200 Upper Valley Pike, Springfield, OH USA

**Keywords:** Biofuel, Pretreatment, Enzymatic hydrolysis, Deacetylation, Mechanical refining, Disk refining, No acid pretreatment, Clean sugar production

## Abstract

**Background:**

A novel, highly efficient deacetylation and disk refining (DDR) process to liberate fermentable sugars from biomass was recently developed at the National Renewable Energy Laboratory (NREL). The DDR process consists of a mild, dilute alkaline deacetylation step followed by low-energy-consumption disk refining. The DDR corn stover substrates achieved high process sugar conversion yields, at low to modest enzyme loadings, and also produced high sugar concentration syrups at high initial insoluble solid loadings. The sugar syrups derived from corn stover are highly fermentable due to low concentrations of fermentation inhibitors. The objective of this work is to evaluate the economic feasibility of the DDR process through a techno-economic analysis (TEA).

**Results:**

A large array of experiments designed using a response surface methodology was carried out to investigate the two major cost-driven operational parameters of the novel DDR process: refining energy and enzyme loadings. The boundary conditions for refining energy (128–468 kWh/ODMT), cellulase (Novozyme’s CTec3) loading (11.6–28.4 mg total protein/g of cellulose), and hemicellulase (Novozyme’s HTec3) loading (0–5 mg total protein/g of cellulose) were chosen to cover the most commercially practical operating conditions. The sugar and ethanol yields were modeled with good adequacy, showing a positive linear correlation between those yields and refining energy and enzyme loadings. The ethanol yields ranged from 77 to 89 gallons/ODMT of corn stover. The minimum sugar selling price (MSSP) ranged from $0.191 to $0.212 per lb of 50 % concentrated monomeric sugars, while the minimum ethanol selling price (MESP) ranged from $2.24 to $2.54 per gallon of ethanol.

**Conclusions:**

The DDR process concept is evaluated for economic feasibility through TEA. The MSSP and MESP of the DDR process falls within a range similar to that found with the deacetylation/dilute acid pretreatment process modeled in NREL’s 2011 design report. The DDR process is a much simpler process that requires less capital and maintenance costs when compared to conventional chemical pretreatments with pressure vessels. As a result, we feel the DDR process should be considered as an option for future biorefineries with great potential to be more cost-effective.

**Electronic supplementary material:**

The online version of this article (doi:10.1186/s13068-015-0358-0) contains supplementary material, which is available to authorized users.

## Background

Successful development, deployment, and commercialization of biochemical processes to convert lignocellulosic biomass into fuels and chemicals are largely dependent on the profitable production of products. Despite the many proposed biological and catalytic conversion pathways to produce fuels (alcohols and hydrocarbon fuels) or chemicals [furfural, 5-hydroxymethylfurfural (HMF), succinic acid, 3-hydroxy propionic acid, levulinic acid, etc.] from biomass, sugars, especially monomeric sugars, are the critical starting substances for most all of the pathways. Therefore, producing low-cost sugars from biomass is critical to the success of a thriving biorefining industry. While the cost of renewable biofuels is heavily impacted by the market price and availability of biomass feedstocks, the primary drivers for sugar production costs from lignocellulosic biomass are the processes and efficiencies of biomass pretreatment and enzymatic hydrolysis. In 2011, NREL generated a TEA model for estimating intermediate sugar production costs that reported a MSSP (i.e., $/lb) for a process that utilized dilute acid pretreatment, solid/liquid separation, and the concentration of the sugar syrups up to 50 wt% without further purification [1]. The model is helpful for the biofuels industry (mainly for non-ethanol producers) as a tool to compare feedstocks and pretreatment process costs [1].

Beyond sugar costs, the production costs of the final fuel and chemical products derived from biomass sugars are still the ultimate criteria to be evaluated for the economic feasibility of a biorefinery process. For a biomass to ethanol process, this criteria is the plant-gate price of ethanol, known as the MESP, which helps policymakers and other stakeholders assess the cost competitiveness and market penetration potential of cellulosic ethanol in comparison with petroleum-derived fuels, and first-generation starch- and sugar-based ethanol [1]. The MESP is highly dependent on both the sugar production costs and the ethanol fermentation yields. High ethanol yields enabled by almost complete utilization of six carbon (C6) and five carbon (C5) sugars in ethanol co-fermentations are essential to make cellulosic ethanol viable. However, complete utilization of C6 and C5 sugars is greatly inhibited by toxic components introduced, or generated during pretreatment, i.e., furfural, HMF, acetate, ammonia, and sulfate [2]. Therefore, providing low-cost, high-concentration sugar syrups with low levels of toxic inhibitors at high process yields is critical for achieving a competitive MESP.

**Fig. 1:**
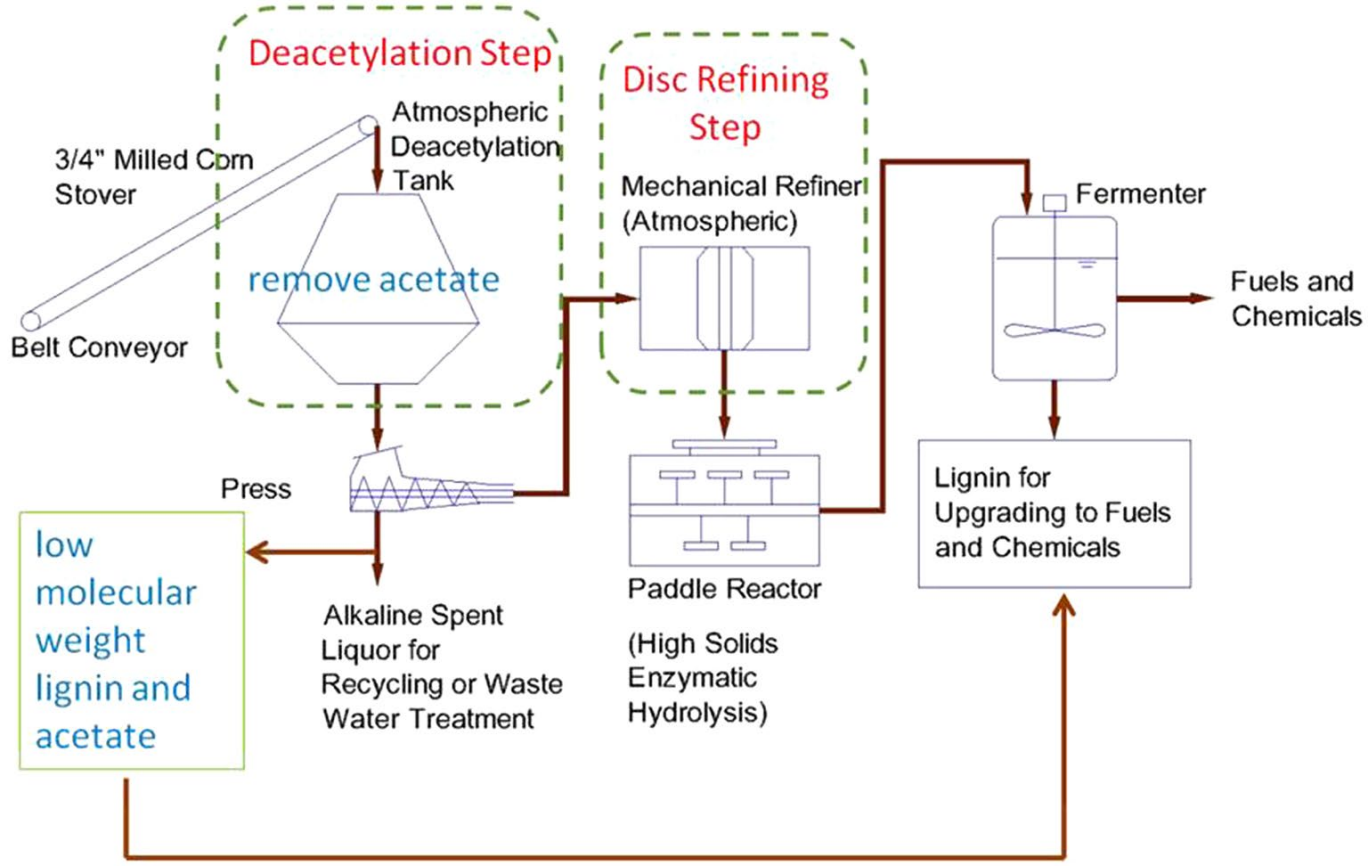
Schematic process flow diagram of deacetylation and disk refining (DDR) process

In a recent publication, we described a novel, simple process consisting of dilute alkali deacetylation followed by mechanical refining in a disk refiner followed by enzymatic hydrolysis (EH) for converting renewable biomass to low-cost sugars at high yields and at high concentrations [3]. Figure 1 shows the schematic process flow diagram of the DDR process. The process features a low-temperature (80 °C), dilute alkaline (40 kg NaOH/ODMT corn stover) deacetylation step followed by mechanical refining in an industrial size disk refiner under modest levels of specific energy consumptions (128–468 kWh/ODMT corn stover). The DDR corn stover residues are subjected to enzymatic hydrolysis under moderate enzyme loadings (17–22 mg total protein per gram of cellulose) at high solids (15 and 20 wt% total insoluble solids) producing monomeric glucose yields ranging from 78 to 84 % and monomeric xylose yields ranging from 71 to 77 %. While high sugar conversion yields are achieved in the DDR process, high concentrations of monomeric sugars are also found exceeding 150 g/L, and total sugars, defined as monomeric sugars plus oligomeric sugars, are found to be greater than 165 g/L. The sugar syrups produced are found with low concentrations (below the detection limits) of the known fermentation inhibitors furfural and HMF. In addition, acetic acid is found to be less than 0.3 g/L. These results suggest that this process has promising applications in various biorefineries [3].

The objective of this work was to evaluate the economic feasibility of the proposed DDR process concept through two criteria: MSSP and MESP. Refining energy and enzyme loadings are the two major process variables in the DDR process which directly affect sugar and ethanol yields. These variables are also the major cost drivers for MSSP and MESP. Therefore, the effects of refining energy and cellulase and hemicellulase loadings on MSSP and MESP are studied through TEA models that incorporate experimental results that were generated from both pilot-scale (deacetylation and disk refining) and bench-scale (high solids enzymatic hydrolysis and fermentation) experimental results. The refining energy and enzyme loading were varied using a response surface methodology (RSM), producing statistically valid empirical equations predicting sugar and ethanol yields. The predicted yields are used in the Aspen Plus process model and the techno-economic model to generate the corresponding MSSP and MESP values as a function of refining energy and enzyme loadings.

## Results and discussion

### Disk refining and enzymatic hydrolysis experimental design using RSM

Experiments based on a response surface, central composite design were carried out to generate empirical models of monomeric glucose yield and monomeric xylose yield as a function of disk refining energy, and cellulase (Novozymes CTec 3) and hemicellulase (Novozymes HTec 3) loadings. The experimental design, data analysis, and model creation were performed in StatEase Design Expert software (version 8.0). Table 1 shows the experimental design conditions. All points were duplicated, while the center point was performed with six replicates to ensure that the model had sufficient power to produce a statistically significant result.

**Table 1:** Disk refining and enzymatic hydrolysis experimental design conditions

Factor	(−) Level	(+) Level	(−) Axial	(+) Axial	Center point
Refining energy (kWh/ODMT)	212	408	128	468	317
CTec 3 (mg t.p./g.o.c.)	15.0	25.0	11.6	28.4	20.0
HTec 3 (mg t.p./g.o.c.)	1.0	4.0	0.0	5.0	2.5

*mg t.p./g.o.c.* mg total protein per gram of cellulose

### Validation of experimental enzymatic hydrolysis yield results

An economical biomass to ethanol process at the commercial scale requires enzymatic hydrolysis to be performed at high solids loadings (15 % or higher), which is essential to reduce water requirements and to increase product titers, reduce the size of enzymatic hydrolysis and fermentation reactors, and reduce the amount of energy needed to purify the ethanol fuel product by distillation [4]. However, calculating sugar yields in high solids enzymatic hydrolysis is more complex, as compared to low solids experiments [5]. In addition, previous experience has shown that even a 1 % deviation in the total solids measurement at the start of enzymatic hydrolysis will cause up to a 10 % deviation in the final sugar yields. Therefore, component mass balances were used to investigate the validity of the sugar yield calculations under high solids enzymatic hydrolysis conditions for selected samples. For these samples, compositional analysis was performed on the solids remaining after enzymatic hydrolysis to measure unconverted cellulose and hemicellulose.

Table 2 shows the solids compositional analysis of the native, deacetylated and disk-refined corn stover (DDR CS) substrate. The native corn stover contains approximately 36 % glucan, 31 % xylan and 3 % acetyl groups. Deacetylation hydrolyzed and removed approximately 80 % of the acetyl groups, 10 % of the xylan and 2 % glucan was solubilized along with 30 % of the lignin and 80 % of the ash. The DCS substrate contains approximately 43 % glucan, 33 % xylan, 13 % lignin, and 0.3 % acetyl groups. Table 2 also shows the compositional analysis of the solid residues after enzymatic hydrolysis at various run conditions corresponding to the run numbers shown in Table 3. The composition of the solids is used in the calculations to determine component mass balance closures.

**Table 2:** Chemical compositions of DDR corn stover biomass before and after enzymatic hydrolysis

	Ash	Lignin	Glucan	Xylan	Galactan	Arabinan	Acetyl
Native CS	2.3 (0.1)^a^	14.9 (0.0)	36.4 (0.0)	30.8 (0.4)	1.8 (0.0)	3.4 (0.0)	2.7 (0.2)
Starting DDR CS	0.6 (0.2)	12.6 (0.4)	43.6 (0.1)	33.1 (0.1)	1.4 (0.0)	2.5 (0.1)	0.3 (0.2)
Run 8 EH residue	0.9 (0.1)	41.1 (0.3)	24.1 (0.0)	22.3 (0.1)	1.26 (0.0)	2.2 (0.0)	0.5 (0.0)
Run 17 EH residue	0.9 (0.0)	34.6 (0.1)	28.0 (0.1)	25.3 (0.1)	1.3 (0.0)	2.3 (0.0)	0.4 (0.0)
Run 22 EH residue	0.8 (0.1)	41.4 (0.7)	24.3 (0.6)	21.9 (0.5)	1.3 (0.0)	2.2 (0.0)	0.4 (0.0)
Run 30 EH residue	1.2 (0.1)	46.3 (0.4)	21.2 (0.2)	19.5 (0.1)	1.4 (0.0)	2.3 (0.0)	0.4 (0.0)
Run 34 EH residue	1.0 (0.1)	45.2 (0.1)	21.9 (0.1)	20.2 (0.2)	1.3 (0.0)	2.2 (0.0)	0.4 (0.0)

^a^Numbers in the parentheses present ± one standard deviation (duplicate samples were used in calculations)

*EH* enzymatic hydrolysis

**Table 3:** Sugar yields after enzymatic hydrolysis of DDR CS substrates

Run number	Refining energy (kWh/ODMT)	CTec 3 (mg/g)	HTec 3 (mg/g)	Monomeric glucose yield (%)	Monomeric xylose yield (%)
1	128	20.0	2.5	79	69
2	212	25.0	4.0	82	78
3	408	25.0	4.0	84	77
4	317	20.0	0.0	80	72
5	408	25.0	1.0	83	74
6	317	20.0	2.5	80	73
7	317	20.0	5.0	84	79
8	317	20.0	2.5	82	76
9	408	15.0	1.0	80	69
10	317	11.6	2.5	78	71
11	408	15.0	4.0	83	73
12	317	11.6	2.5	80	73
13	128	20.0	2.5	77	67
14	317	20.0	2.5	81	74
15	317	28.4	2.5	84	79
16	408	25.0	1.0	83	74
17	408	15.0	1.0	77	66
18	212	25.0	1.0	80	74
19	317	28.4	2.5	87	81
20	317	20.0	2.5	80	72
21	212	15.0	4.0	79	73
22	317	20.0	2.5	81	75
23	317	20.0	0.0	79	70
24	212	25.0	1.0	81	75
25	212	25.0	4.0	83	78
26	212	15.0	1.0	76	68
27	212	15.0	4.0	80	74
28	317	20.0	5.0	82	76
29	468	20.0	2.5	83	78
30	468	20.0	2.5	84	77
31	408	15.0	4.0	84	76
32	212	15.0	1.0	78	70
33	317	20.0	2.5	83	77
34	408	25.0	4.0	87	80

**Fig. 2:**
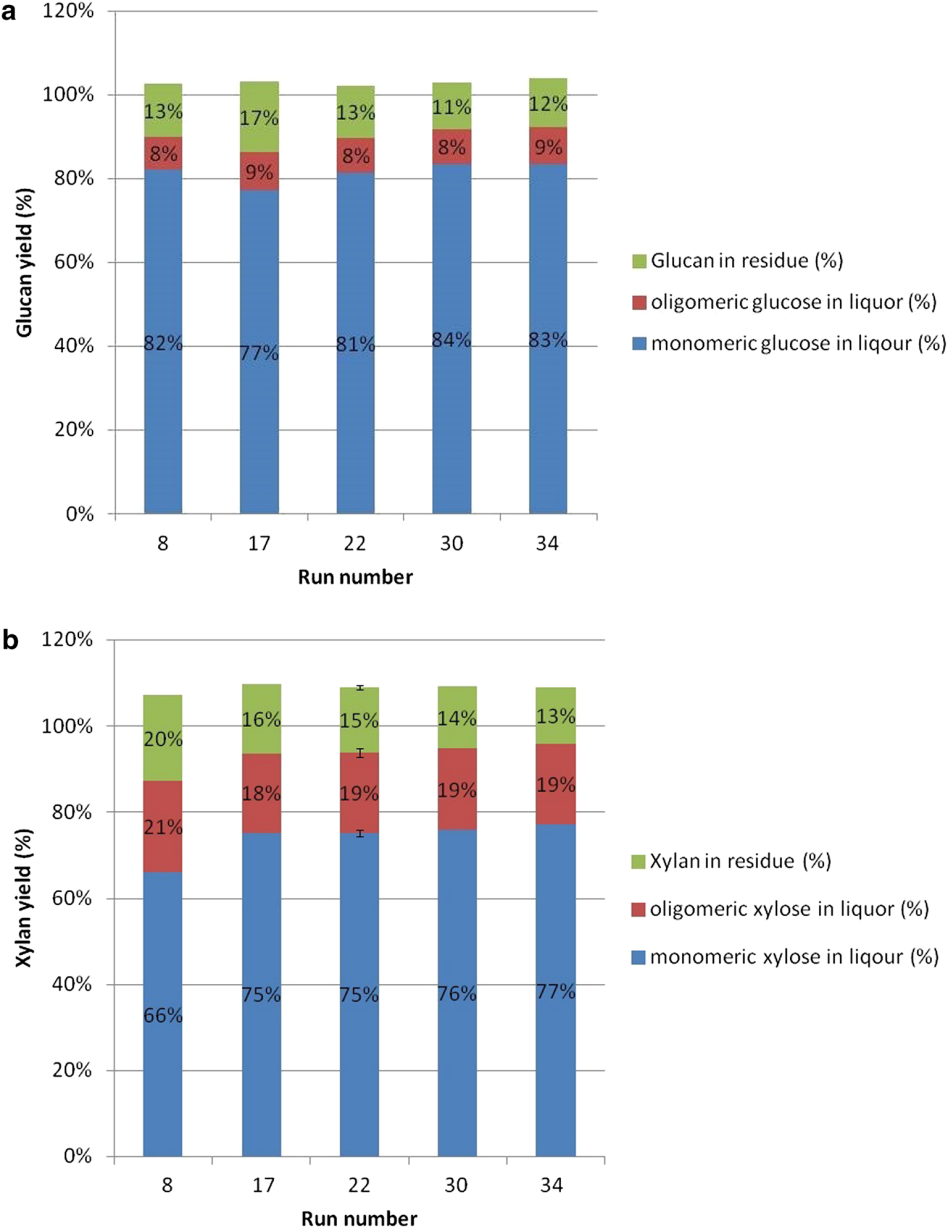
Glucan mass balance closures after enzymatic hydrolysis **a** Glucan mass balance; **b** Xylan mass balance

Figure 2a shows glucan mass closure for several selected samples. The corresponding treatment and enzymatic hydrolysis conditions can be found according to the run numbers in Table 3. The fraction of original glucan present as insoluble glucan content after enzymatic hydrolysis varied between 11 and 17 % depending on the refining and enzymatic hydrolysis conditions. The monomeric glucose yield was also sensitive to both conditions, as the difference in yields in these selected samples was about 7 %, while the oligomeric glucose yields were consistently between 8 and 9 %, regardless of refining energy and enzyme loading variations. For all five selected samples, the glucan mass closures ranged from 102 to 103 %. The consistently 2–3 % higher over 100 % glucan mass closure is most likely due to the overestimation of the hydrolyzed oligomeric glucose using the Sugar Recovery Standards (SRS) method in liquor compositional analysis [6]. The SRS method basically corrects sugar degradation during acid hydrolysis using monomeric sugar standards. This method may result in overestimation of oligomeric sugars as polymeric sugars have slower degradation kinetics [7]. Although imperfect, the glucose yields calculated from these experiments are acceptable for use in the Aspen Plus and RSM models.

Figure 2b shows xylan mass closure for the same set of samples. The fraction of original xylan present as insoluble xylan content after enzymatic hydrolysis varied between 13 and 20 %, while the monomeric xylose yields were in the range from 66 to 77 %. The oligomeric xylose yields hydrolyzed during enzymatic hydrolysis were consistently between 18 and 21 %. Once again, the overall xylan mass closures were consistently high in between 107 and 109 %, suggesting a stronger tendency to overestimate xylo-oligomers than gluco-oligomers. It is because xylose degrades much faster than glucose under the same acid condition, whereas xylo-oligomers are more resistant to acid degradation. However, the overestimation of xylo-oligomers will not affect the final techno-economic analysis in current study because oligomeric sugars cannot be converted to ethanol during fermentation. Thus, the yields of oligomeric sugars are not used in the Aspen plus modeling nor in the economic analysis.

### Constructing and validating the design of experiment (DoE) model

The energy levels and enzyme loadings selected for the DoE are considered to span the area of practical interest to the biorefinery industry that could produce economically viable yields. For example, we chose an enzyme loading of 20 t.p. mg/g.o.c (total protein mg per gram of cellulose, 1 mg t.p./g.o.c is approximately 0.75 FPU) as used in NREL’s 2011 design report for the bioethanol platform [1]. According to the economic modeling results, refining energies over 500 kWh/ODMT would result in a huge demand for the importation of electricity. In addition, refining input energy is inversely related to the biomass throughput of a disk refiner. In practice, the higher refining energy input is often realized by lowering the biomass feeding rate and the clearance in between the two refining plates to extend the residence time of the biomass in the disk refiner. Thus, a process relying on high energy consumption levels in disk refining will not only suffer an increase in electrical operational costs, but will also have elevated capital requirements, as more disk refiners are required. Therefore, high operational costs and capital investments become significant issues for disk refining processes with refining energy requirements over 500 kWh/ODMT.

Table 3 shows the experimental conditions used as input for the TEA model, as well as the sugar yields from enzymatic hydrolysis. Monomeric glucose and xylose yields ranged between 76–87 % and 66–81 %, respectively.

The empirical linear models (applicable only within the ranges tested) for monomeric glucose and xylose yields are as follows:

1 $${\text{Glucose Yield }} = { 67}. 6 6 4 { } \,+ \, 0.0 1 5 { } \times {\text{ Refining Energy }} + \, 0. 3 4 7 { } \times {\text{ CTec3 }} + \, 0. 8 6 4 { } \times {\text{ HTec3}}$$

2 $${\text{Xylose Yield }} = { 57}. 2 7 4 { } + \, 0.0 1 2 { } \times {\text{ Refining Energy}} + \, 0. 4 6 5 { } \times {\text{ CTec3 }} + { 1}. 4 6 8\; \times {\text{ HTec3}} .$$

**Fig. 3:**
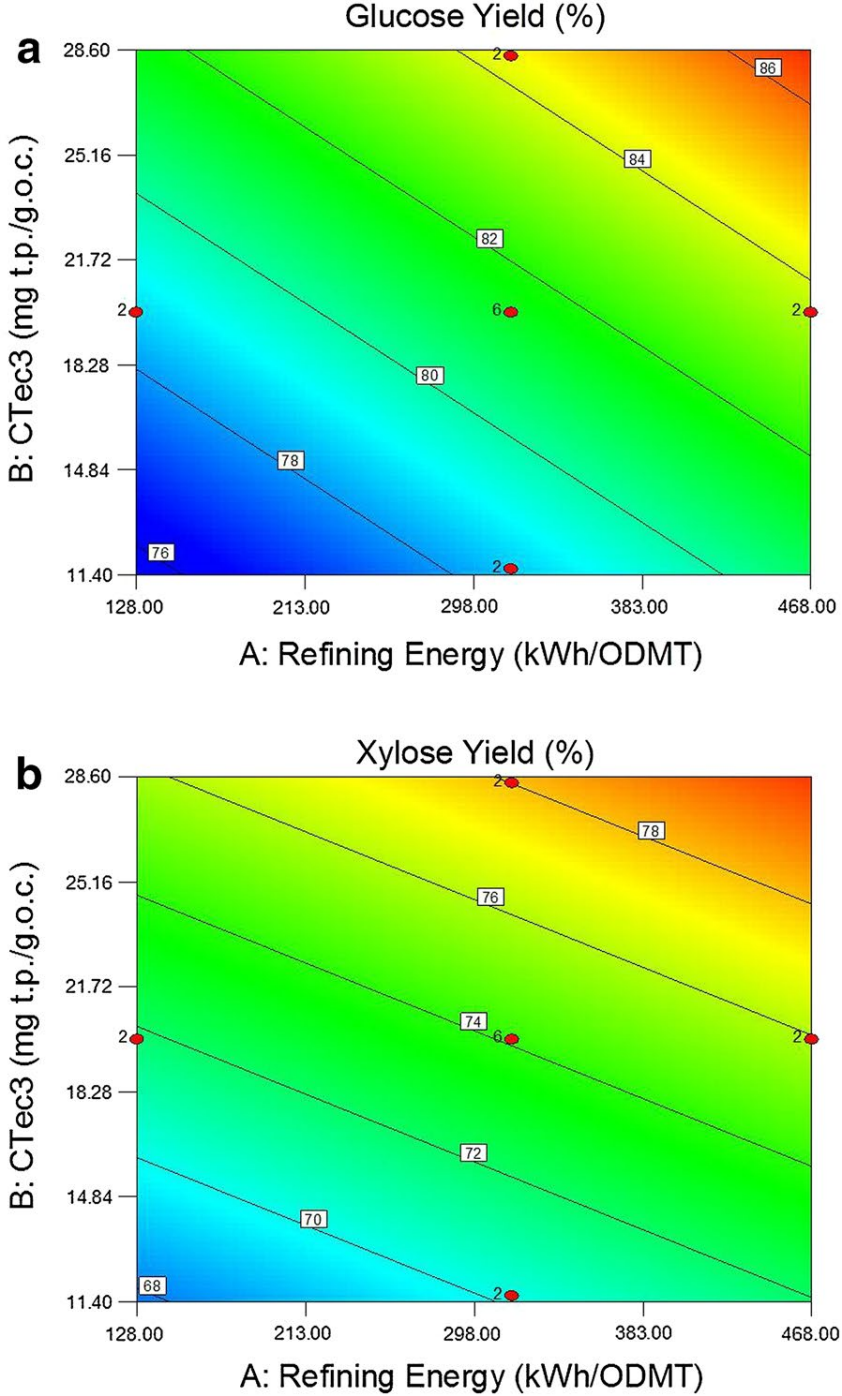
Effect of refining energy on glucose yields (**a**) and xylose yields (**b**) (Actual factor: HTec3 loading of 2.5 mg t.p./g.o.c.)

The adequacy of the developed linear models is examined through an analysis of variance (ANOVA). Additional file 1: Table S1 shows the corresponding “*p* values” for both models. The Model *F* Values of 43.49 for the glucose model and 25.41 for the xylose model imply that both models are significant. There is only a 0.01 % chance that a “Model *F* Value” this large could occur due to noise. Values of “Prob > *F*” less than 0.05 indicate model terms are significant. All three model terms: refining energy, CTec3 loading and HTec3 loading are significant at a 95 % confidence level because *p* values are less than 0.05. The models indicate that the glucose and xylose yields are positively linearly correlated to disk refining input energy and enzyme loadings within the limited ranges examined in this study. There were no statistically significant two-factor or quadratic interactions observed within the dataset. The relationships between refining energy, CTec3 loading, and HTec3 loadings on glucose and xylose yields and corresponding response surfaces are shown in Fig. 3a, b, respectively. The linear model suggests that higher sugar yields will occur at higher refining energy and enzyme loadings. The coefficient of determination, *R*^2^, is 0.81 and 0.72 for monomeric glucose and xylose yields, respectively, suggesting the glucose and xylose are linearly correlated with refining energy and enzyme loadings but not perfectly described by the model.

### Aspen plus model and economic analysis

The Aspen plus model used in current study was adopted and modified from 2012 NREL’s bioethanol platform design report based on the dilute acid pretreatment. In the modified model, low-temperature deacetylation tank (price quoted for stainless steel reactor with temperatures up to 130 °C) replaced the high-temperature (typically 150–190 °C) horizontal screw-type reactors constructed with expensive metal alloys (i.e. Incoloy clad), resulting in a nearly $29 million savings in capital investment for pretreatment reactors.

In the Aspen plus model, deacetylation was modeled at a solid to liquid ratio of 1:3 followed by washing using the same amount of water. The excessive deacetylation liquor and washing liquor were separated from the biomass solids to attain 40 % total solids in facilitating the subsequent disk refining and high solids enzymatic hydrolysis. In practice, this separation, similar to acid impregnation and dewatering, was done using screw-type presses. Thus, the OPEX and CAPEX of the solid liquid separation remained the same as acid pretreatment for the deacetylation in the model. The pressed liquor containing lignin, acetate and spent chemicals was acidified to precipitate lignin used for boiler fuels while the rest was sent to the wastewater treatment. In all, it is estimated that for every gallon of ethanol produced from the DDR process approximately 3.5–4.5 gallon of fresh water was needed. This design, though very similar to dilute acid pretreatment, needs further investigation and optimization on water recycling and sodium recovery to make dilute alkaline pretreatment more cost-effective and environmental-friendly.

The simulation of disk refining in Aspen plus model was realized using a calculation box to incorporate the milling energy consumptions. The DDR process eliminates the large steam demand required for high-temperature dilute acid pretreatment. However, it also requires a large amount of electricity to power the disk refiners. To calculate the electricity cost, two assumptions based on electricity price are used in the economic sensitivity analysis presented here. Electricity is sold to the grid at $0.06/kW when there is an excess and is purchased at $0.08/kW imported price when there is a demand [1, 8, 9]. As shown in Additional file 1: Table S2, the biorefinery ethanol process with refining energy at 128 kWh/ODMT could be self-sustaining when lignin is burned using a combined heat and power scenario. At mechanical refining input energies greater than 317 kWh/ODMT, the importation of electricity from the grid is needed regardless of enzyme loadings. However, with mechanical refining input energies between 128 and 317 kWh/ODMT, the amount of electricity needed for the DDR process is dependent on enzyme loadings, because of the power required for air compressors for aerobic enzyme production. For example, with refining energy consumption at 212 kWh/ODMT, electricity purchases are required at higher enzyme loading because more electricity is required for aeration of on-site cellulase enzyme production tanks.

It is noted that all enzymatic hydrolysis experiments were performed at 15 wt% total insoluble solids (TS), which is lower than the 20 % TS used in previous work [1, 8]. However, the 15 wt% insoluble TS studied here contains 15 wt% insoluble solids (IS) content which is comparable to a 20 wt% TS slurry produced by dilute acid pretreatment due to solubilization of most of the hemicellulose. It is commercially desirable to perform enzymatic hydrolysis at higher TS loadings to reduce energy consumption in the distillation step. In the future, we will explore enzymatic hydrolysis performance at 20 % up to 30 % insoluble TS to determine the economic benefits of operating at higher solids loadings. In addition, the estimated enzyme costs in this study of $4.24/kg of protein are not representative of Novozymes current or future pricing, but were based on TEA models described in the 2011 NREL’s design report, that in turn were based on an nth plant, corn stover biomass to ethanol process at the 60 million gallon/year plant nameplate design throughput, incorporating on-site enzyme production using pure glucose as the carbon source [1], and in no way reflects current or future costs provided by the enzyme vendors.

Refining energy consumption not only affects the operational costs by increasing overall process electricity demand, but also impacts the number of disk refiners required for a 2000 ODMT/day cellulosic biorefinery. Each industrial-scale 56/60-inch (142–152 cm) disk refiner was quoted at roughly $2.3 M installed capital. For instance, three industrial-scale 56/60” single disk refiners were found to be required for a 2000 ODMT/day facility at the 128 kWh/ODMT refining energy. Increasing refining energy increases the refining residence time, leading to lower production rates per disk refiner unit. Therefore, more disk refiners are needed to meet the same feedstock throughput. For the high energy case modeled here, we found a requirement of nine disk refiners is needed to provide the refining energy of 468 kWh/ODMT for the 2000 ODMT/day facility. The results of the TEA model are generated based on the monomeric sugar yields shown in Table 3. On the fermentation side, the DDR corn stover hydrolyzates are highly fermentable due to the low concentrations of inhibitors such as acetic acid and furfural. Therefore, glucose-to-ethanol and xylose-to-ethanol yields used in the TEA are both set at 90 % based on previous experimental results for fermentation of corn stover from DDR treatment and enzymatic hydrolysis [3]. As shown in Additional file 1: Table S2, regression analysis is used for generating models for empirical prediction of net electricity demand, ethanol yield, MSSP and MESP as a function of refining energy and enzyme loadings. The number of disk refiners are determined by the refining energy, as used in the previous section. Net electricity (shown as $/gal in Additional file 1: Table S2) is calculated from the integrated process models with negative values for importing and positive values for exporting electricity. The ethanol yields (gal/ODMT corn stover feedstock), MSSP and MESP are all calculated using TEA models with adjustments of the input variables (enzyme loadings, refining energies and yields) for each run.

As shown in Additional file 1: Table S2, the highest experimental ethanol yield of 89 gallon/ODMT is found when the refining energy is 317 kWh/ODMT and the enzyme loading used was 28.4 mg CTec3 protein/g of cellulose and 2.5 mg HTec3 protein/g of cellulose, the highest enzyme loading used in this experimental design. The lowest ethanol yield is found to be 77 gallon/ODMT corn stover at the lowest enzyme loadings of 15 mg CTec3 protein/g of cellulose and 1 mg HTec3 protein/g of cellulose.

The best fit to the experimental results are linear regression models as follows:

3 $${\text{Ethanol Yield }}\left( {{\text{gal}}/{\text{tonne}}} \right) = { 66}. 9 5 1\,+ 0.0 1 4\; \times {\text{ Refining Energy }} + \, 0. 4 2 9\; \times {\text{CTec3}} + 1. 1 8 1 { } \times {\text{ HTec3}}$$

4 $${\text{Electricity demand }}\left( {\$ /{\text{gallon of ethanol}}} \right) \, = \, - 2 2. 7 9 1+ 0.0 6 2\; \times \;{\text{Refining Energy}} + 0. 3 80\; \times \;{\text{CTec3}} + 0. 7 2 5\; \times {\text{ HTec3}}$$

The linear models are also examined for their adequacy using ANOVA analysis. Additional file 1: Table S2 shows the “Model *F* Value” and values of “Prob > *F*” for both the empirical model of ethanol yield and electricity demand, respectively, showing both models to be adequate. Meanwhile, ethanol yield is somehow well described by the model with *R*^2^ equal to 0.79, while electricity demand displays a strong linear correlation (*R*^2^ = 0.99) with the variables investigated.

**Fig. 4:**
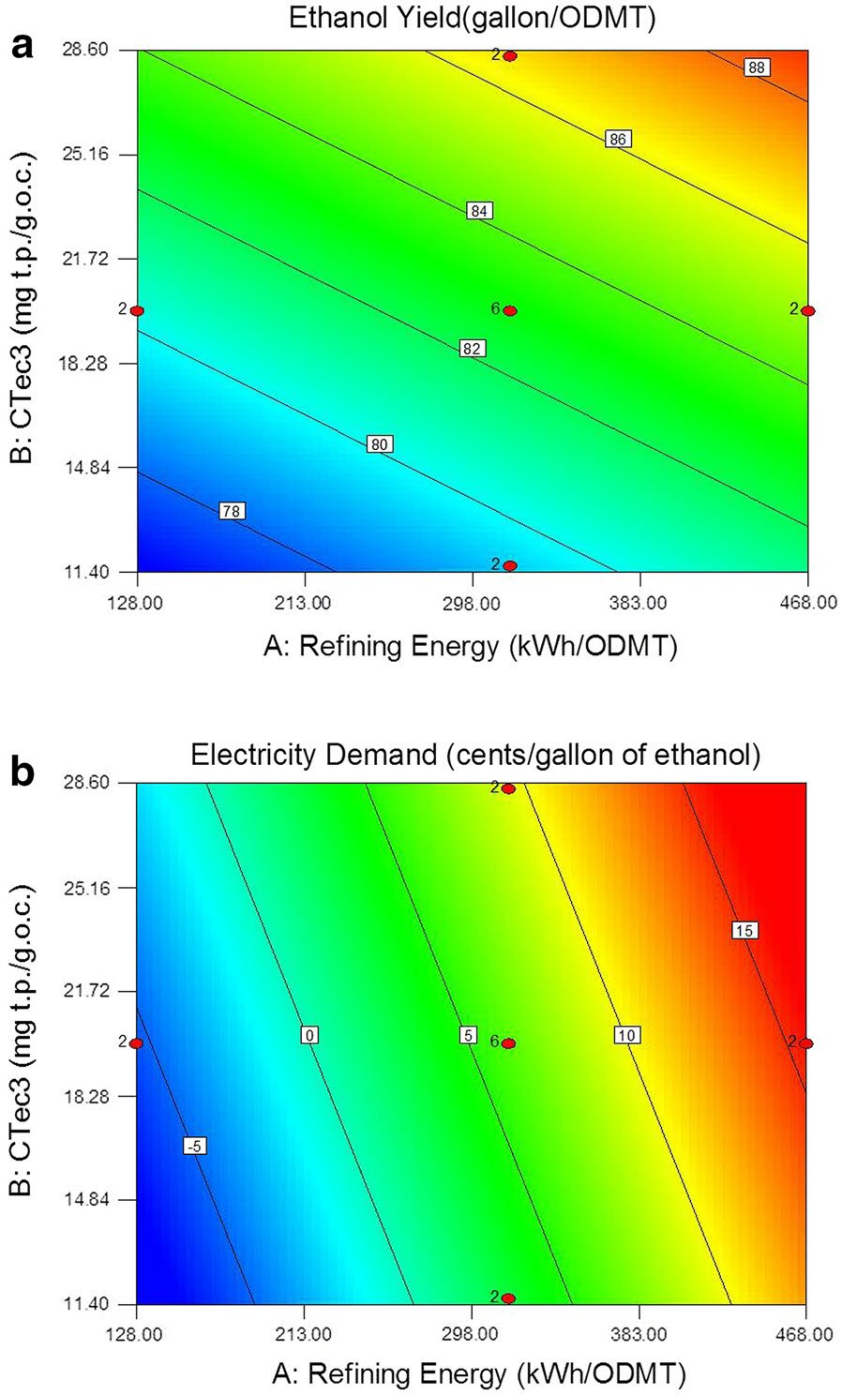
Effect of refining energy and enzyme loadings on ethanol yields (**a**) and electricity demand (**b**). (HTec3 = 2.5 mg t.p./goc)

Figure 4 shows the effect of refining energy and enzyme loadings on ethanol yield and electricity demand. It would be incorrect to assume ethanol yield will increase beyond the range of variable values explored in this study at higher refining energy and enzyme loadings, since the level of biomass recalcitrance to enzymes significantly increases after approximately 80 % or more of the easily digestible cellulose is hydrolyzed. In addition, ethanol yield is also restricted by the initial carbohydrate content in the biomass. As shown in Fig. 4a, ethanol yield is linearly correlated to glucose and xylose yields, ranging between approximately 75 and 90 gallons per metric tonne of corn stover. The higher ethanol yields that occurred at higher levels of refining energy and enzyme loading could increase revenue by up to approximately $35/metric tonne of corn stover (assuming $2.30 per gallon of ethanol), but may also lead to a higher operational cost, particularly with regard to electricity demand.

The effect of refining energy and enzyme loading on electricity demand is plotted in Fig. 4b. The electricity demand also displays a positive linear relationship with refining energy and enzyme loadings, ranging from approximately −7 to 20 cents/gallon of ethanol. As a point of comparison, a bioethanol process using conventional acid pretreatment will show exporting electricity at a revenue of approximately 10.8 cents/gallon of ethanol according to the NREL 2011 bioethanol platform design report [1]. It is noteworthy that this result is based on an acid–steam pretreatment at 160 °C for approximately 10 min followed by enzymatic hydrolysis at an enzyme loading of 20 mg t.p./g.o.c.. The DDR process replaces steam energy usage with mechanical refining energy. The results shown in Fig. 4 imply refining at energy input of 128 kWh/ODMT is approximately equivalent to the steam energy consumption used in the design report and would likely maintain an electricity export of 10 cents/gallon of ethanol. However, if the refining energy exceeds 200 kWh/ODMT, the electricity produced from lignin combustion will be consumed within the process, and the importation of electricity is required.

### Effect of refining energy and enzyme loading on MSSP and MESP

The MSSP and MESP are calculated based on the economic analysis methodology described in the methods section and predicted using regression analysis. Linear regression models for MSSP and MESP are found to have better adequacy as compared to other possible models (e.g., a Quadratic model), as shown by the corresponding *p* values shown in Additional file 1: Table S4. The “Lack of Fit *F* value” of 1.57 for MSSP and 3.63 for MESP implies the Lack of Fit is not significant relative to the pure errors. The *R*^2^ value for MSSP and MESP is 0.55 and 0.74, respectively.

The linear regression models for MSSP and MESP are as follows:

5 $${\text{MSSP }} = \, 0. 1 9\,+\, 2. 8 1 {\text{E}} - 0 5 { } \times {\text{ Refining Energy}} \,+\, 5. 1 4 {\text{E}} - 0 4 { } \times {\text{ CTec3}} - 1. 2 3 {\text{E}} - 0 3 { } \times {\text{ HTec3}}$$

6 $${\text{MESP }} = { 2}.0 5\,+\, 5.0 4 {\text{E}} - 00 4 { } \times {\text{ Refining Energy }} \,+ \, 0.0 1 { } \times {\text{ CTec3 }} - \, 0.0 1\; \times {\text{ HTec3}}$$

**Fig. 5:**
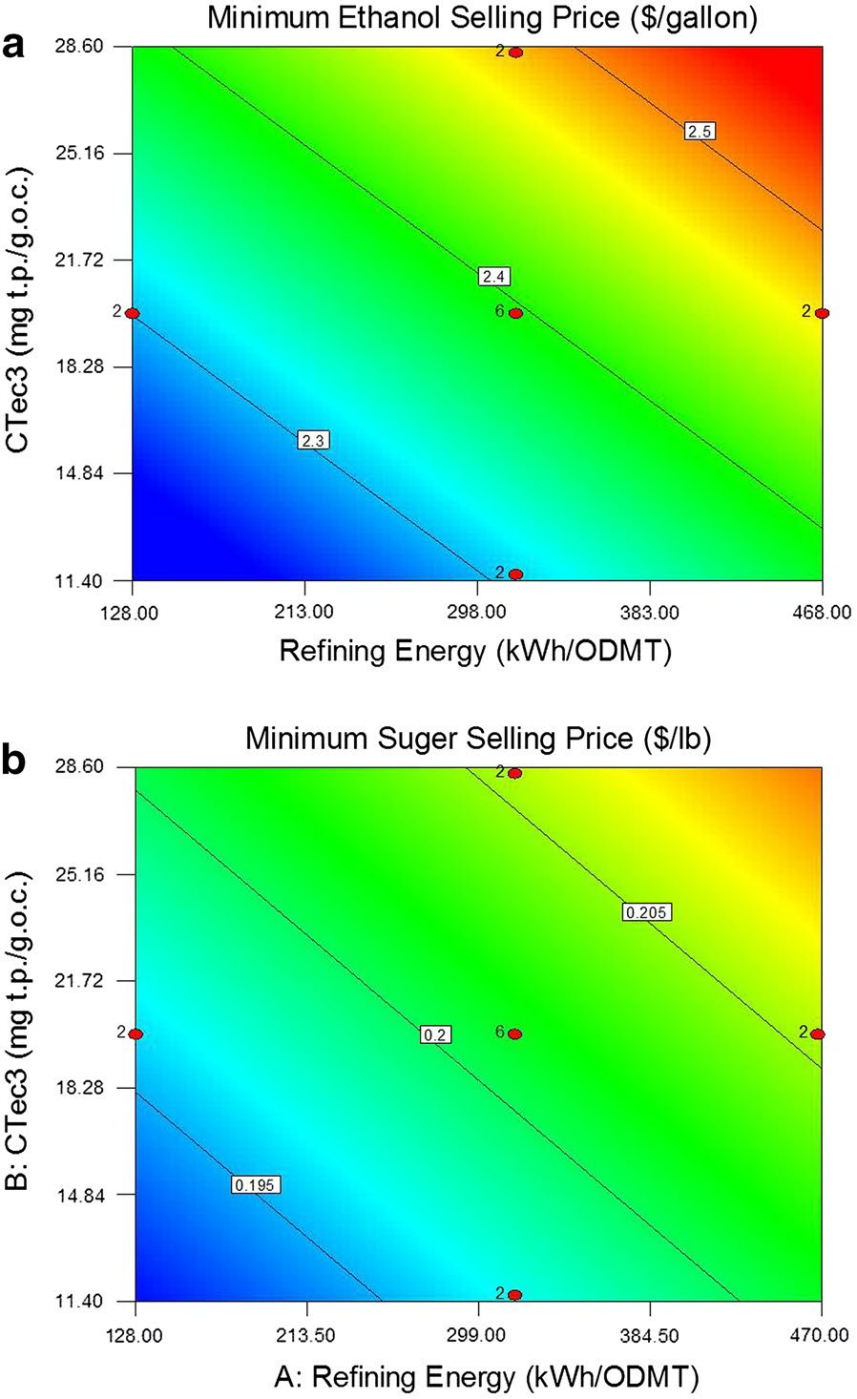
Effect of refining energy and enzyme loadings on MESP (**a**) and MSSP (**b**) (HTec3 = 2.5 mg t.p./goc)

MSSP and MESP are both positively correlated to refining energy and CTec3 loading, but inversely correlated to HTec3 loading, as illustrated in Fig. 5. Higher refining energy and CTec3 loadings lead to higher MSSP and MESP within the boundaries of the experimental conditions due to higher electricity demand and increased number of disk refiner units. The model also suggests that the cost of increased HTec3 loadings over the current loading range is not offset by higher ethanol yields and the associated increase in revenue. The MESP ranges from $2.24/gal to $2.54/gal, close to the $2.15/gal ethanol reported in the 2012 state-of-technology (SOT) demonstration using dilute acid pretreatment [10]. It should be noted that the enzymatic hydrolysis and fermentation for the 2012 study were performed and modeled at 20 wt% TS, while it is at 15 wt% TS in this study. Potential economic benefit with higher TS is not yet considered in the MESP or MSSP, and will be addressed in the future. The MESP could be further reduced if sugar yield can be further improved while not increasing the refining energy input or enzyme loading. This requires an improved refining effect, which could be achieved by redesigning the disk plates, or integrating a secondary refiner, such as a low consistency disk refiner or Szego mill. We are currently engaged in ongoing research at NREL exploring these options that will be addressed in future publications.

From 2011 to 2014, the market price of raw sugar fluctuated between $0.180 and $0.284/lb [11]. The MSSP of the novel DDR process ranges from $0.191 to $0.212/lb, suggesting that the use of DDR sugar is likely viable for biofuels and chemical production. The DDR sugar syrups, containing approximately 20–40 % five carbon sugars and the remainder 6 carbon sugars, are very low in acetic acid, furfural and HMF content relative to many other hydrolysate liquors from lignocellulosic pretreatments. When compared to sugar syrups derived from sulfuric acid or ammonia pretreatments, the DDR hydrolyzates are low in sulfur or ammonia content, respectively. Thus, DDR hydrolysate sugars offer improved fermentability and lower toxicity to both biocatalysts and noble metal catalysts used to upgrade the sugars to intermediates suitable for upgrading to hydrocarbon fuels.

Therefore, with the potential for process improvements and optimization, the DDR process may become a process alternative with high potential to replace dilute acid pretreatment. The ability to produce cleaner and possibly less-expensive hydrolyzate sugars, the novel DDR process may be amenable to processes being utilized to produce drop-in hydrocarbon fuels and chemicals [3].

## Conclusion

In this work, the highly efficient DDR process concept was evaluated for economic feasibility through techno-economic analysis. Two major cost-driven parameters, refining energy and enzyme loadings, were investigated in the TEA model and analysis. Experiments were first performed to gather the model-required data, and then the results from the experimental design covering the most reasonable operating conditions were used in the TEA model. The yield calculations were validated by showing good cellulose and hemicellulose mass balance closures. Sugar and ethanol yields showed positive linear correlation to refining energy and enzyme loadings with good adequacy. However, the corresponding MSSP and MESP at higher refining energy and enzyme loadings also escalated in a linear relationship, implying that the marginal increase of ethanol yield could not justify the elevated energy and enzyme costs. The MSSP and MESP of the DDR process were found in a similar range as found for a deacetylation followed by dilute acid pretreatment process modeled in the NREL 2011 design case. The lowest MSSP, $0.1913/lb of sugar, and MESP, $2.24/gallon of ethanol, are achieved at two experimental conditions: (1) refined at 212 kWh/ODMT and hydrolyzed with an enzyme loading of 19 mg t.p./g.o.c (2) refined at 317 kWh/ODMT and hydrolyzed with an enzyme loading of 14.1 mg t.p./g.o.c. Considering that the novel DDR process is much simpler and requires much less maintenance, it should be considered as an option for going forward into a biorefinery industry with great potential to be more cost-effective.

## Methods

### Materials

Corn stover was harvested in 2009 in Hurley County, South Dakota, United States, and transported to the Idaho National Laboratory, where it was stored indoors and hammer milled to pass a 2” round screen. It was shipped to NREL in January 2013. Upon receipt at NREL, the corn stover was knife milled (Jordan Reduction Solutions, Birmingham, AL, USA) to pass through a 19-mm (0.75 inch round holes) screen and stored indoors in 200 kg lots in supersacks.

### Pilot-scale deacetylation and disk refining

Corn stover deacetylation was performed in a 1900-L paddle mixer (American Process Systems, Gurnee, IL, USA). Dry corn stover (100–120 dry kg) was added to the paddle mixer along with a dilute 0.1 M sodium hydroxide solution. The 8 % (w/w) total solids slurry was heated to 80 °C and held for 2 h, and then the liquor was allowed to drain overnight through screens (2 mm openings) located in the bottom ports of the paddle mixer. The solids were mixed with additional water and then the rinse water was drained through 20-mesh screens in ports on the bottom of the mixer. The solids were pumped to a continuous screw press (Vincent Corp. Model CP10, Tampa, Florida, United States) for dewatering to between 45 and 50 % (w/w) total solids. Nine batches of deacetylated corn stover (1000 kg total) were prepared in this manner, sealed in plastic bags, loaded into 55-gallon drums, and shipped to the Andritz R&D facility in Springfield, OH, USA, for mechanical refining in their Sprout Model 401 36-inch (91 cm) commercial-scale disk refiner. The atmospheric refiner trials were conducted at five different feed mass flow rates. The Sprout model 401 refiner has two counter rotating disks, each driven by 225 kW (300 hp) motors. For the studies reported here, the refiner rotational speed was maintained at 1200 rpm. A Durametal 36,104 plate pattern consisting of a fine-bar design formulated for fiber strength development in pulping was the configuration of the rotating plates used in the Sprout 401 refiner. The feed material was weighed and then fed to the refiner via a conveyor. The goal was to maximize the refiner motor load for the given feed mass flow rate. The energy consumption varied from 128 to 468 kWh/ODMT.

### Bench-scale enzymatic hydrolysis

The enzymatic cellulose digestibility of deacetylated corn stover (DCS) treated by the disk refiner was measured at high solids conditions (15 wt% insoluble solids). Hydrolysis was conducted with 60 g of slurry in 250-mL capped Schott media bottles. The bottles were autoclaved empty, and then the pH-adjusted, disk-refined substrates were manually introduced into the bottles using a small funnel to reach the target total solids concentration of 15 or 20 % (w/w). Two mL of citrate buffer (pH 5.1, 1.0 M) was added to each flask to help maintain pH at approximately 5.0. Enzymatic hydrolysis began by adding enzyme to achieve the target enzyme dosages as shown in Table 3, then placing the fully loaded and capped bottles in a shaking incubator operating at 150 rpm and 48 °C for 4 days. A National Institute of Standards and Technology (NIST)-certified thermometer (Thermo Scientific, Waltham, MA, USA) was used to verify shaker incubator temperature. Duplicate flasks were performed for all enzyme loadings. The experiments were run for 4 days, with time course samples taken once daily. Time zero concentration values were calculated based on composition of the pretreated slurry and then adjusted based on the weight additions of water, citrate buffer, and enzyme. Final samples were taken at day four and analyzed for density, and total and insoluble solids, as well as monomeric and oligomeric total sugar concentrations. The density and solids composition measurements were completed using NREL’s standard laboratory analysis methods [12]. Cellulose conversion yield calculations during enzymatic hydrolysis were calculated from the net amount of monomeric glucose produced, which also used measurements of liquid density and liquid volume.

### Experimental design

A central composite design (CCD) was used to characterize the reaction space with regard to enzyme loading levels, and the level of refining input energy. The design used in this experiment was a 2-level, 3-factor factorial with a center point (replicated 6 times) and six axial points (replicated 2 times), with the axial points specified to maintain full rotatability. The calculation of the axial points, specification of the design, and randomization of the experimental design was done using Design-Expert 8.0 (Stat-Ease, Minneapolis, MN, USA), but was consistent with the methodology described by Meyers, Montgomery, and Cook [13]. The selection of experimental conditions was based on a range of previous studies and is described in Table 3.

Regression analysis was performed with Design-Expert 8.0 to generate empirical models of whole process cellulose-to-glucose and xylan-to-xylose yields, ethanol yield per metric tonne of dry corn stover, MSSP, and MESP. The software was used to determine which factors from a linear, quadratic, or two-factor interaction model were significant at a 95 % confidence level (*p* value of 0.05 or less) using one-way ANOVA analysis. Factors with the largest *p* values were progressively removed from each respective model until only significant variables remained.

### Techno-economic analysis

The TEA model developed for this paper included a conceptual process design using a process flow diagram, detailed process modeling for rigorous calculation of the material, and energy balances using Aspen Plus [1]. A simplified block flow diagram of the bioethanol platform used in the Aspen plus model is shown in Additional file 1: Figure S1. In all, the process is divided into nine areas including: Feed Handling (A100), Pretreatment and Conditioning (A200), Enzymatic Hydrolysis and Fermentation (A300), Enzyme Production (A400), Distillation and Solids Separation (A500), Wastewater Treatment (A600), Storage (A700), Burner/Boiler Turbogeneration (A800) and Utilities (A900). This model originally developed based on dilute acid pretreatment process was modified to enable the simulation of DDR pretreatment process by mainly substituting the dilute acid pretreatment unit operations in A200 using the DDR process partially described in Fig. 1. The remaining areas of the model inherited most of the original designs from the 2012 NREL design report including using both deacetylation extracted lignin and enzymatic hydrolysis lignin residues as the source of boiler fuel. The potential cost benefit from wastewater reduction of DDR process by reducing sulfuric acid and ammonia hydroxide usage was not incorporated in the model due to the lack of experiment data on wastewater evaluation.

The resulting capital investment, project, and operating cost estimates were translated into discounted cash flow calculations. From this information, a MSSP and a MESP were established based on a stipulated 10 % internal rate of return (IRR). The OPEX calculation for the designed facility was based on material and energy balance calculations using Aspen Plus process simulations [14]. Raw material unit costs were based on literature or existing models, summarized in the 2011 NREL cellulosic ethanol design report [1]. Major raw materials included sulfuric acid, di-ammonium phosphate, ammonia, corn steep liquor, purchased sugar for enzyme production, water, and cooling tower chemicals. All costs were inflated to 2011 US dollars using the Plant Cost Index from Chemical Engineering Magazine [11–15], the Industrial Inorganic Chemical Index from SRI Consulting [16], and the labor indices provided by the US Department of Labor Bureau of Labor Statistics [17]. Salaries for personnel were inflated from 2009 dollars to 2011 dollars. Ninety percent of the total salaries are added for labor burden, and 3.0 % of the inside boundary limit (ISBL) capital expenses was designated for maintenance. Property insurance and taxes accounted for 0.7 % of the Fixed Capital Investment.

Material, energy balance, and flow rate information was used to size equipment based on the Aspen Plus simulation of the material and energy balances. CAPEX was calculated from equipment cost obtained from vendor quotations, prior published NREL design reports [1, 10], or from internal equipment costing databases. For most equipment, scaling factors were applied for variations in the throughput or other key design parameters relative to the original design basis using standard methodologies as described in the NREL 2002 and 2011 design cases [1, 18].

The discounted cash flow assumed 40 % equity financing with a loan interest at 8 % for 10 years. Working capital was assumed to be 5 % of the fixed capital investment. The plant depreciation period was set for 7 years. The plant was assumed to take 3 years to construct with a quarter of a year spent on start up. The MSSP and the MESP were the price at which sugar or ethanol must be sold to reach an IRR of 10 %.

The purpose of cost analysis for sugar was merely to separate the cost of producing sugars from the downstream costs of producing ethanol or other products. The sugar and ethanol TEA work is based on the design and models discussed in our previous work with the following changes [1]: (1) all process design through enzymatic hydrolysis was kept the same for the sugar model. (2) A lignin press with counter-current washing was added after hydrolysis to separate lignin and unreacted insoluble solids from the dilute mixed sugar stream [1]. (3) A triple-effect evaporator system was added to the model, with heat input specified to achieve 50 % water in the sugar syrup. The MSSP was calculated using dry weight sugar basis, although the sugar syrup product contained 50 % water and other non-sugar compounds that may require further cleanup, at additional cost.

### Analytical methods

The composition of the corn stover solids was determined by a two-stage acid digestion procedure based on NREL standard Laboratory Analysis Procedure (LAP No. NREL/TP-510-42627) [19]. Soluble sugars, acetic acid, and degradation products were determined by NREL LAP No. NREL/TP-510-42623 [6].

The density of liquid samples was measured using an Anton-Parr model DMA-500 density meter (Anton Parr USA, Inc., Ashland, VA, USA).

## Additional file

10.1186/s13068-015-0358-0 Additional infomation on design of experiments and process simulation using Aspen plus.

## Abbreviations

TEA: techno-economic analysis
DDR: deacetylation and disk refining process
MSSP: minimum sugar selling price
MESP: minimum ethanol selling price
NREL: National Renewable Energy Laboratory
ODMT: oven dried metric tonne
HMF: 5-hydroxymethylfurfural
RSM: response surface methodology
DoE: design of experiment
DOE: Department of Energy
ANOVA: analysis of variance
CS: corn stover
DCS: deacetylated corn stover
mg t.p./g.o.c.: mg total protein per gram of cellulose
TS: total solids
IS: insoluble solids
CCD: central composite design
NIST: National Institute of Standards and Technology
ISBL: inside boundary limit
IRR: internal rate of return
CAPEX: capital expenditures
OPEX: operational expenditures
