# Supplementary material for: Techno-economic analysis of the deacetylation and disk refining process: characterizing the effect of refining energy and enzyme usage on minimum sugar selling price and minimum ethanol selling price
Source: Biotechnol Biofuels. 2015 Oct 29;8:173. doi: 10.1186/s13068-015-0358-0 (PMC4625976; doi:10.1186/s13068-015-0358-0)
Supplement: Supplementary file 1 — 10.1186/s13068-015-0358-0 Additional infomation on design of experiments and process simulation using Aspen plus. [file 13068_2015_358_MOESM1_ESM.docx]

**Table S1. Statistical analysis of the effects of disc refining and enzymatic hydrolysis parameters on monomeric glucose and xylose yield**

|  | Monomeric glucose yield | | Monomeric xylose yield | |
| --- | --- | --- | --- | --- |
| R^2^ | 0.8131 | | 0.7176 | |
| Model F value | 43.49 | | 25.41 | |
| Prob.>F | <0.0001(significant) | | <0.0001(significant) | |
| Terms | F value | *p* value ( Prob > F) | F value | *p* value ( Prob > F) |
| Specific energy of disc refining | 40.81 | <0.0001(significant) | 9.52 | 0.0043(significant) |
| CTec3 loading | 57.54 | <0.0001(significant) | 35.18 | <0.0001(significant) |
| HTec3 loading | 32.12 | <0.0001(significant) | 31.52 | <0.0001(significant) |
| Lack of fit | 0.87 | 0.5793 (not significant) | 2.91 | 0.0199 (significant) |

**Table S2. Effect of operational variables and capital variables on ethanol yield, MSSP, and MESP**

| Run Number | Operational Variables | | | Capital Variables | Responses | | | |
| --- | --- | --- | --- | --- | --- | --- | --- | --- |
|  | Refining energy (kWh/  ODMT) | CTec 3 (mg/g) | HTec 3 (mg/g) | Number of Disc Refiners | Net Electricity Demand (cents/gal of ethanol) | Ethanol Yield (gal/ O.D. ton) | MSSP ($/lb) | MESP ($/gal) |
| 1 | 128 | 20.0 | 2.5 | 3 | -5.9 | 79.19 | $0.1988 | $2.34 |
| 2 | 212 | 25.0 | 4.0 | 4 | 2.8 | 85.10 | $0.1995 | $2.39 |
| 3 | 408 | 25.0 | 4.0 | 8 | 13.6 | 85.70 | $0.2096 | $2.54 |
| 4 | 317 | 20.0 | 0.0 | 6 | 5.1 | 81.20 | $0.2016 | $2.39 |
| 5 | 408 | 25.0 | 1.0 | 8 | 12.2 | 83.80 | $0.2102 | $2.53 |
| 6 | 317 | 20.0 | 2.5 | 6 | 5.7 | 81.60 | $0.2042 | $2.43 |
| 7 | 317 | 20.0 | 5.0 | 6 | 8.5 | 86.66 | $0.1966 | $2.35 |
| 8 | 317 | 20.0 | 2.5 | 6 | 6.9 | 84.10 | $0.1985 | $2.36 |
| 9 | 408 | 15.0 | 1.0 | 8 | 8.9 | 79.70 | $0.2041 | $2.42 |
| 10 | 317 | 11.6 | 2.5 | 6 | 3.1 | 79.60 | $0.1964 | $2.30 |
| 11 | 408 | 15.0 | 4.0 | 8 | 11.1 | 83.28 | $0.2009 | $2.39 |
| 12 | 317 | 11.6 | 2.5 | 6 | 4.00 | 81.60 | $0.1913 | $2.24 |
| 13 | 128 | 20.0 | 2.5 | 3 | -7.5 | 77.20 | $0.2038 | $2.39 |
| 14 | 317 | 20.0 | 2.5 | 6 | 6.1 | 82.70 | $0.2015 | $2.40 |
| 15 | 317 | 28.4 | 2.5 | 6 | 9.7 | 86.65 | $0.2053 | $2.48 |
| 16 | 408 | 25.0 | 1.0 | 8 | 12.3 | 83.80 | $0.2100 | $2.53 |
| 17 | 408 | 15.0 | 1.0 | 8 | 7.8 | 76.67 | $0.2123 | $2.51 |
| 18 | 212 | 25.0 | 1.0 | 4 | 0.9 | 82.10 | $0.2019 | $2.40 |
| 19 | 317 | 28.4 | 2.5 | 6 | 10.6 | 89.26 | $0.1996 | $2.41 |
| 20 | 317 | 20.0 | 2.5 | 6 | 5.5 | 81.20 | $0.2054 | $2.44 |
| 21 | 212 | 15.0 | 4.0 | 4 | -1.1 | 81.10 | $0.1937 | $2.27 |
| 22 | 317 | 20.0 | 2.5 | 6 | 6.4 | 83.10 | $0.2005 | $2.39 |
| 23 | 317 | 20.0 | 0.0 | 6 | 4.4 | 79.70 | $0.2047 | $2.43 |
| 24 | 212 | 25.0 | 1.0 | 4 | 1.2 | 83.10 | $0.1996 | $2.37 |
| 25 | 212 | 25.0 | 4.0 | 4 | 3.3 | 85.63 | $0.1986 | $2.37 |
| 26 | 212 | 15.0 | 1.0 | 4 | -3.8 | 77.00 | $0.1987 | $2.31 |
| 27 | 212 | 15.0 | 4.0 | 4 | -0.4 | 82.09 | $0.1913 | $2.25 |
| 28 | 317 | 20.0 | 5.0 | 6 | 7.6 | 84.14 | $0.2021 | $2.42 |
| 29 | 468 | 20.0 | 2.5 | 9 | 15.8 | 85.63 | $0.2042 | $2.46 |
| 30 | 468 | 20.0 | 2.5 | 9 | 15.8 | 85.72 | $0.2040 | $2.45 |
| 31 | 408 | 15.0 | 4.0 | 8 | 11.5 | 85.30 | $0.1965 | $2.34 |
| 32 | 212 | 15.0 | 1.0 | 4 | -2.7 | 79.10 | $0.1937 | $2.26 |
| 33 | 317 | 20.0 | 2.5 | 6 | 7.2 | 85.20 | $0.1962 | $2.34 |
| 34 | 408 | 25.0 | 4.0 | 8 | 14.6 | 88.80 | $0.2028 | $2.46 |

**Table S3. Statistical analysis of the effects of disc refining and enzymatic hydrolysis parameters on ethanol yield and electricity demand**

|  | Ethanol yield | | Electricity demand | |
| --- | --- | --- | --- | --- |
| R^2^ | 0.79 | | 0.99 | |
| Model F value | 37.84 | | 767.75 | |
| Prob.>F | <0.0001(significant) | | <0.0001(significant) | |
| Terms | F value | *p* value ( Prob > F) | F value | *p* value ( Prob > F) |
| Specific energy of disc refining | 22.87 | <0.0001(significant) | 2046.31 | 0.0043(significant) |
| CTec3 loading | 53.84 | <0.0001(significant) | 193.37 | <0.0001(significant) |
| HTec3 loading | 36.80 | <0.0001(significant) | 63.57 | <0.0001(significant) |
| Lack of fit | 1.46 | 0.2242 (not significant) | 1.94 | 0.0979 (significant) |

**Table S4. Statistical analysis of the effects of disc refining and enzymatic hydrolysis parameters on MSSP and MESP**

|  | MSSP | | MESP | |
| --- | --- | --- | --- | --- |
| R^2^ | 0.5533 | | 0.7375 | |
| Model F value | 12.38 | | 28.1 | |
| Prob.>F | <0.0001(significant) | | <0.0001(significant) | |
| Terms | F value | *p* value (prob>F) | F value | *p* value (prob>F) |
| Specific energy of disc refining | 16.31 | 0.0003(significant) | 38.83 | <0.0001(significant) |
| CTec3 loading | 13.77 | 0.0008(significant) | 41.82 | <0.0001(significant) |
| HTec3 loading | 7.08 | 0.0124(significant) | 3.63 | <0.0662 (not significant) |
| Lack of fit | 1.57 | 0.1870 (not significant) | 3.63 | 0.1228 (not significant) |


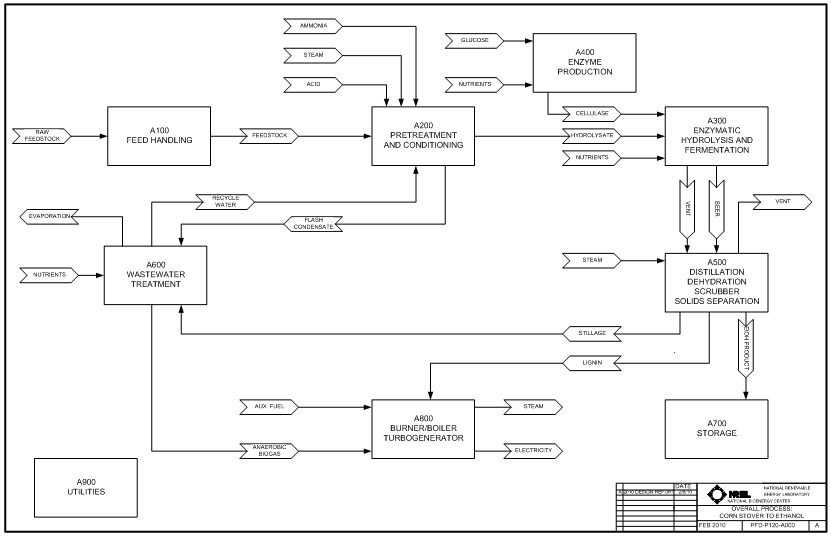
Figure S1. Schematic diagram of bioethanol platform simulated in Aspen plus process model
